# Advancing the management of prosthetic joint infections: a review of randomized controlled trials and emerging evidence

**DOI:** 10.1128/aac.00338-25

**Published:** 2025-08-14

**Authors:** Burcu Isler, Zhenya Welyczko, Nicholas Jorgensen, Joshua Davis, David L. Paterson

**Affiliations:** 1 University of Queensland Centre for Clinical Research 303224, Brisbane, Australia; 2 Department of Infectious Diseases, Gold Coast University Hospital 60093 , Gold Coast, Australia; 3 Department of Orthopaedic Surgery, Princess Alexandra Hospital 1966, Brisbane, Australia; 4 Infection Research Program, Hunter Medical Research Institute, University of Newcastle 454568, New Lambton Heights, Australia; 5 ADVANCE-ID, Saw Swee Hock School of Public Health and Yong Loo Lin School of Medicine, National University of Singapore 203377 , Singapore, Singapore; Houston Methodist Hospital and Weill Cornell Medical College, Houston, Texas, USA

**Keywords:** prosthetic joint infection, clinical trials, antibiotics, surgical management

## Abstract

Prosthetic joint infections (PJI) remain among the most devastating complications in orthopedic surgery, with increasing incidence paralleling the growth in arthroplasty procedures worldwide. While treatment protocols are well-established, evidence supporting current approaches is lacking, and outcomes remain suboptimal, highlighting the need for improved therapeutic strategies. The objective of this study is to review ongoing and recently completed randomized controlled trials (RCTs) investigating novel approaches to PJI prevention and treatment. We searched PubMed and ClinicalTrials.gov databases for RCTs that focused on PJI management, and that are ongoing or completed within the 6 months preceding February 28, 2025. We included investigator-initiated trials, studies of novel therapeutic agents, and studies on infection prevention strategies. We extracted and reviewed data regarding trial design, interventions, and outcomes. We identified significant advances in three key areas: (i) investigator-initiated trials exploring optimization of current surgical and antimicrobial treatment strategies, including the ROADMAP adaptive platform trial (ii); novel therapeutic agents, including engineered antibacterial peptides, monoclonal antibodies, and new-generation antibiotics specifically targeting biofilm-associated infections; and (iii) prevention strategies, particularly focusing on perioperative antibiotic protocols. Recent trials demonstrate promising approaches to reducing antibiotic exposure while maintaining efficacy, novel mechanisms for biofilm disruption, and strategies for optimizing perioperative prophylaxis. Investigator-initiated trials like ROADMAP and SOLARIO are challenging traditional paradigms, particularly in antibiotic duration, while novel therapeutics targeting biofilm through various mechanisms show promise in early-phase trials. Future research should prioritize cost-effectiveness analyses, targeted studies for specific patient subgroups, and evaluation of combination therapeutic approaches.

## INTRODUCTION

Prosthetic joint infections (PJI) represent one of the most challenging complications in orthopedic surgery, with reported incidence rates of 1%–2% for primary arthroplasties and significantly higher rates for revision procedures (1). Despite being relatively uncommon, their impact is profound—PJI are associated with mortality rates of 8%–25% within 5 years and represent the most common cause of failure in total joint arthroplasty (2). Moreover, current approaches to PJI management demonstrate failure rates ranging from 11% to 35% (3). The economic burden is substantial, with annual treatment costs in Australia exceeding $250 million, based on an average hospital cost of $64,585 per patient (4). Looking forward, the combined annual hospital costs related to PJI of the hip and knee in the United States are projected to reach $1.85 billion by 2030 (5).

The complexity of PJI management stems from multiple factors. The biofilm on implant surfaces, increasing antimicrobial resistance, and the diversity of causative organisms all contribute to treatment challenges (6). Current standard treatments, whether through debridement, antibiotics, and implant retention (DAIR) or staged revision procedures, show variable success rates and often require prolonged antibiotic therapy with associated toxicities and complications (7).

Traditional treatment approaches have largely evolved through observational studies and expert consensus, with relatively few high-quality randomized controlled trials (RCTs) informing clinical practice (8). Recent clinical trials range from large-scale investigations of established treatment paradigms to studies of novel therapeutic agents targeting specific aspects of PJI pathophysiology.

This review aims to provide a comprehensive analysis of ongoing and recently completed RCTs in PJI management. We focus on three key areas: investigator-initiated trials examining current treatment strategies, studies of novel therapeutic agents, and trials of prevention protocols. Our objective is to critically evaluate these emerging approaches and their potential impact on clinical practice.

## METHODOLOGY

We conducted a systematic search of PubMed using the terms (“prosthetic joint infection” OR “periprosthetic joint infection” OR “PJI”) AND (“randomized controlled trial” OR “RCT” OR “clinical trial”) AND (“surgical management” OR “antibiotic therapy”). In addition, we searched ClinicalTrials.gov using the terms “prosthetic joint infection” OR “periprosthetic joint infection.” Studies were included if they were:

1. Randomized controlled trials of Phase 1b or later
2. Focused on PJI prevention or treatment
3. Ongoing or completed within the 6 months preceding February 28, 2025 (i.e., 1/9/2024–28/2/2025)

We extracted data regarding trial design, intervention characteristics, primary and secondary outcomes, current status, and preliminary or final results where available.

## INVESTIGATOR-INITIATED TRIALS

### ROADMAP trial: Randomised Arthroplasty Infection Worldwide Multidomain Adaptive Platform Trial

The ROADMAP trial, formally titled the “Randomised Arthroplasty Infection Worldwide Multidomain Adaptive Platform trial,” is an Australia-led multicenter, pragmatic, adaptive platform trial with planned enrollment of up to 2,500 participants across Australia, New Zealand, Canada, and the United Kingdom (9).

The trial will evaluate several key treatment strategies: DAIR versus revision arthroplasty for late acute PJI (PJI presenting more than 30 days post-implant and with a short duration of symptoms), 6 versus 12 weeks of antibiotic therapy following single-stage revision, extended versus no antibiotic prophylaxis after second-stage revision, and rifampicin-containing versus non-rifampicin containing antibiotic regimens for Gram-positive infections managed with DAIR. For context, there are three main surgical management strategies for the treatment of PJI, the most common one being DAIR, where the infected prosthesis is retained, but undergoes extensive debridement and irrigation, as well as exchange of any easily removable parts such as polyethylene liners. The more traditional curative strategy is two-stage revision, in which the infected prosthesis is completely removed, a temporary cement spacer is placed in the joint space and is retained for 6–12 weeks, and a new prosthesis is inserted at a second operation. An alternative revision strategy is a one-stage revision, where the infected prosthesis is removed, the joint space is debrided, and a new prosthesis is inserted all at the same operation (9).

Patient eligibility requires confirmed or likely PJI of a large joint according to European Bone and Joint Infection Society (EBJIS) 2021 criteria, physical presence at a participating hospital during eligibility assessment, and current PJI with active symptoms or signs. Confirmed PJI as per EBJIS criteria requires the presence of at least one highly specific criterion, such as a sinus tract communicating with the prosthesis or two or more positive cultures with phenotypically identical organisms. If these are absent, the diagnosis may be considered likely if there is a combination of suggestive findings (e.g., elevated synovial leukocyte count, positive histology, or a single positive culture) in the appropriate clinical context. Cases that do not meet these criteria are classified as unlikely to be infected.

Exclusion criteria include previous ROADMAP participation for the same joint, previous ROADMAP participation for different joints within 12 months, non-curative treatment intent, inability to be classified into defined silos, and inaccessibility for 12-month follow-up (9).

For the rifampicin versus no rifampicin domain, patients are randomized to receive a backbone antibiotic regimen either with or without oral rifampicin. The backbone regimen is determined by the treating team. Eligibility is assessed between 48 hours and 7 days after initial surgical intervention for infections that are either culture-negative or caused by Gram-positive bacteria. Patients are excluded if they have known rifampicin hypersensitivity, significant drug-drug interactions, documented rifampicin resistance, or are pregnant or breastfeeding.

For the extended versus no antibiotic prophylaxis after second-stage revision domain, randomization occurs after reimplantation between extended antibiotic prophylaxis (12 weeks) versus no additional prophylaxis, contingent on negative intraoperative cultures obtained during the second-stage procedure.

For the 6 versus 12 weeks of antibiotic therapy following the first-stage revision domain, participants will receive 6 or 12 weeks (±7 days) of antibiotics following single-stage surgery.

The route and choice of antibiotics are determined by the treating clinician for all domains. All domains exclude patients with fungal infections, difficult-to-treat bacterial pathogens, or those requiring planned long-term antibiotic suppression therapy.

The primary outcome measure is treatment success at 12 months post-platform entry, defined by a composite of patient survival, clinical cure (i.e., no clinical or microbiological evidence of ongoing infection), discontinuation of antibiotics for the index joint, and retention of the prosthesis inserted after initial management. Secondary outcomes include a desirability of outcome ranking (DOOR) that orders participants based on survival, joint function, treatment success, and quality of life. Patient-reported outcomes comprise Oxford Hip Score or Oxford Knee Score for joint function and EQ-5D-5L for quality of life, both assessed at 12 months. Economic evaluation employs Bayesian simulation techniques to estimate incremental cost-effectiveness ratios and net monetary benefits, incorporating treatment costs, surgical expenses, readmissions, and quality-adjusted life years. Additional outcomes include individual primary endpoint components, microbiological relapse and reinfection rates, and time to revision surgery captured through national joint replacement registry linkage.

The trial recruited the first patient in March 2025 and will continue recruitment through at least December 2028 (Table 1).

**TABLE 1:** Investigator-initiated trials in PJI management^*a*^

Trial name	Phase	Surgical strategy	Intervention	Status	Key outcome measure	Key results	Reference
ROADMAP	3	DAIR vs revision for late acute PJI	Multiple interventions: surgical strategy comparison, 6 vs 12 weeks of antibiotics for single stage, extended vs no prophylaxis post-second stage, rifampicin vs non-rifampicin regimens	First patient recruited in March 2025	Treatment success at 12 months (composite of survival, clinical cure, antibiotic discontinuation, prosthesis retention)	Not yet available	(9)
SOLARIO	3	All surgical strategies with complete source control	Short (≤7 days) vs long (≥4 weeks) systemic antibiotics with local antibiotic therapy for bone and joint infections including PJI	Completed 2024	Treatment failure within 12 months after surgery	Short course non-inferior to long course; better safety profile (82.8% vs 54.1% adverse-event free at 6 weeks). Results are aggregate for all orthopedic infections; PJI-specific results not yet published	(10, 11)
RiCOTTA	3	DAIR only	Monotherapy vs combination therapy with rifampicin	Recruiting (66 patients enrolled as of April 2025)	Treatment success at 15 months after DAIR	Not yet available	(12, 13)
RIFAMAB	3	DAIR only	Rifabutin vs rifampicin for staphylococcal PJI	Recruiting (10/30 sites as of December 2024)	Treatment failure within 1 year	Not yet available	(14)
OPTION	3	One-stage vs two-stage revision	One-stage vs two-stage exchange arthroplasty	Ongoing (completion December 2025)	Treatment success at 1 year (absence of reoperation for PJI)	Interim analysis: 98% vs 94% success; no significant difference between groups	(15–17)
CORGI	2	Two-stage revision (post-reimplantation phase)	Omadacycline vs standard-of-care oral antibiotics for bone and joint infections including PJI	Recruiting (primary completion December 2025)	Treatment success at 2 weeks post-therapy completion	Interim safety data: 59 patients enrolled (only 5% PJI, 88% diabetic foot infections)	(18, 19)
LITE	3	Two-stage revision (LITE procedure)	Short course (2 weeks) vs long course (12 weeks) antibiotics post-reimplantation	Completed	Infection control rate (composite of clinical cure and antibiotic discontinuation) at 24 months	Identical infection control rates (96.7% vs 96.7%); the short course had fewer complications and lower costs	(20)
ProHipQ-OA	3	Primary THA for osteoarthritis	Single vs multiple dose antibiotic prophylaxis in primary THA	Ongoing	PJI incidence at 90 days	Not yet available	(21)
ProHipQ-F	3	Primary THA for fractures	Single vs multiple dose antibiotic prophylaxis in fracture-related THA	Ongoing	PJI incidence at 90 days	Not yet available	(22)
TKA Antibiotic Prophylaxis	3	Primary TKA	Single vs multiple dose antibiotic prophylaxis in elective TKA	Ongoing	PJI incidence at 90 days	Not yet available	(23)

^*a*^ PJI, prosthatic joint infection; DAIR, Debridement, antibiotics, implant retention; THA, total hip arthroplasty; TKA, total knee arthroplasty.

The trial’s adaptive platform design will enable concurrent evaluation of multiple therapeutic strategies across different centers and healthcare systems. As the first large-scale RCT to address these fundamental questions in PJI management, the results from this international study will provide evidence-based data to inform clinical practice guidelines.

### SOLARIO trial: Short or Long Antibiotic Regimens in Orthopaedics

The SOLARIO trial (Short or Long Antibiotic Regimes in Orthopaedics) was a UK-led multicenter, open-label, randomized controlled non-inferiority trial that compared the efficacy of short versus long courses of systemic antibiotic therapy in adults with orthopedic infections treated operatively with local antibiotic therapy (10). This first-of-its-kind study enrolled 500 patients across multiple UK hospitals, including cases of PJI and other orthopaedic infections. Participants were randomized in a 1:1 ratio to receive either a short course (≤7 days) or a currently recommended long course (≥4 weeks) of systemic antibiotics, with the primary outcome being treatment failure within 12 months after surgery.

Results presented at the European Bone and Joint Society’s annual congress (Barcelona, September 26–28, 2024) demonstrated that the short-course group received a median of 5 days of systemic antibiotics compared to 37 days in the long-course group, representing an average reduction of 47 antibiotic days per patient. The cumulative reduction across the study population was 11,275 antibiotic days. Local antibiotic delivery was achieved through bioresorbable products (such as CERAMENT G [BonesSupport] or Osteoset-T [Wright Medical]) in 79% of cases, while bone cement (PMMA) was used in 19% of cases, primarily for patients with PJI (11).

It is important to note that patients treated with DAIR for PJI were excluded from this trial, as surgical source control had to be complete to meet eligibility criteria. PJI treated with revision arthroplasty were eligible.

There was no difference in treatment failure between the groups, confirming the non-inferiority of the shorter regimen. The short-course group demonstrated better safety outcomes. At 6 weeks, 82.8% of patients in the short-course group reported no adverse events, compared to 54.1% in the standard group, with severe adverse events being markedly lower in the short course group, as well (3.9% vs. 20.7%, *P* < 0.0001). This trend continued at 3–6 months, with 91.5% of the short-course group free from adverse events compared to 79.6% in the standard group, and severe events remaining significantly lower in the short course group (4.2% vs. 9.4%, *P* <0.049) (11).

Pending publication of the complete study, which will detail the specific types of surgical infections, these initial results demonstrate that combining surgical source control with local antibiotic therapy in the absence of prolonged systemic antibiotic therapy is an effective and safe approach for selected patients with osteoarticular infection.

### RiCOTTA trial: rifampicin combination versus monotherapy for Staphylococcal PJI

The RiCOTTA trial is a multicenter, open-label, randomized controlled non-inferiority trial comparing oral antimicrobial monotherapy to rifampicin combination therapy in the oral treatment phase of staphylococcal PJI managed with DAIR (12).

The trial is being conducted across 18 hospitals in the Netherlands, including 6 university medical centers and 12 general hospitals, with a target enrollment of 316 patients. Participants include adults aged 18 years or older with confirmed hip or knee PJI according to the EBJIS 2021 definition, where causative organisms are *S. aureus* and/or coagulase-negative staphylococci, managed with DAIR and planned for 12 weeks of antibiotic treatment (13).

Key exclusion criteria include resistance to rifampicin, allergic reactions to rifampicin, or significant drug interactions with rifampicin, as well as contraindications to levofloxacin, clindamycin, cotrimoxazole, and tetracyclines in the form of resistance, hypersensitivity or drug drug interactions. Patients with complicated *S. aureus* bacteremia or concurrent endocarditis requiring intravenous antibiotics for more than 3 weeks are excluded, along with those experiencing treatment failure before oral therapy initiation, more than two separate surgical debridements before the intravenous-to-oral switch, or unsatisfactory response leading to intravenous therapy beyond day 21. Additional exclusions include PJI of tumor or megaprostheses, planned chemotherapy within 12 months, chronic suppressive therapy, pregnancy or breastfeeding, and life expectancy less than 12 months.

Participants are randomized between 1 and 7 days before transitioning from intravenous to oral antibiotics. The rifampicin arm receives rifampicin 450 mg twice daily combined with levofloxacin 500 mg twice daily, while the monotherapy arm receives clindamycin 600 mg three times daily. Alternative regimens are permitted in strict hierarchical order for cases involving antimicrobial resistance, drug unavailability, or polymicrobial infections. For the rifampicin arm, alternatives to levofloxacin include clindamycin, cotrimoxazole, or tetracyclines in that order. For the monotherapy arm, alternatives include cotrimoxazole, levofloxacin, or tetracyclines. The total antibiotic treatment duration is 12 weeks regardless of the assigned regimen.

The primary outcome is treatment success at 1 year after completing antimicrobial therapy (15 months after DAIR). Success is defined as the absence of infection-related repeat surgery, new antibiotic treatment for the index joint, ongoing antibiotic use at follow-up completion, and death. The study assumes an 85% treatment success rate with rifampicin combination therapy and uses a non-inferiority margin of 10%. Secondary outcomes include quality of life measured using the EQ-5D-5L questionnaire, adverse events, development of *Clostridioides difficile* infections during treatment, the number of switches to different oral regimens, and development of rifampicin resistance in patients with confirmed staphylococcal PJI relapses. Patient recruitment commenced in May 2023, with 66 patients enrolled as of April 2025. The trial has an estimated completion date of March 2027.

The results of this trial could significantly impact clinical practice by validating monotherapy without rifampicin as an alternative to rifampicin-based combination therapy, particularly in settings where rifampicin combinations are considered standard treatment for staphylococcal infections. The findings could also support clinical practice in regions where rifampicin is not routinely used, potentially encouraging the adoption of other highly bioavailable oral anti-staphylococcal antibiotics, such as trimethoprim-sulfamethoxazole and tetracyclines. This would be especially beneficial for patients with contraindications to or poor tolerance of rifampicin-based regimens. Together with ongoing research initiatives like the ROADMAP trial, the RiCOTTA study will contribute valuable evidence toward optimizing antimicrobial strategies for PJI management.

### RIFAMAB trial: rifabutin versus rifampicin for Staphylococcal PJI

The RIFAMAB trial is a multicenter, open-label, randomized controlled non-inferiority trial designed to evaluate the effectiveness of rifabutin compared to rifampicin in the treatment of staphylococcal PJI managed with DAIR (14). The study aims to demonstrate the non-inferiority of rifabutin, which has a better safety profile compared to rifampicin, particularly concerning adverse events and drug-drug interactions.

The trial is being conducted across multiple hospitals in France, with a target enrollment of 436 patients. Participants include those with confirmed staphylococcal PJI involving the hip or knee, managed with DAIR. Key inclusion criteria are a confirmed diagnosis of PJI caused by *S. aureus* or coagulase-negative staphylococci (CoNS), with polymicrobial infections excluded. Participants are randomized in a 1:1 ratio to receive either rifabutin (300 mg daily) or rifampicin (10 mg/kg per day, range 600 mg to 1,200 mg daily) in combination with another antibiotic (e.g., penicillin, fluoroquinolone, doxycycline/minocycline, oxazolidinone, cotrimoxazole, daptomycin, glycopeptide, macrolide, fusidic acid) for 12 weeks. The primary outcome is treatment failure within 1 year, defined by the need for further surgical intervention, PJI-related death, or the requirement for suppressive antibiotic therapy that was not planned before randomization. The study started in November 2021 and is anticipated to be completed by June 2027. Ten of the 30 hospitals listed on clinicaltrials.gov are recruiting patients as of December 2024.

While these results will benefit settings that rely on rifamycin for treating staphylococcal PJI, the question remains whether combining rifamycins with antistaphylococcal antibiotics leads to better outcomes than antistaphylococcal antibiotics alone. The ROADMAP and RICOTTA trials aim to answer this question.

### OPTION trial: one-stage versus two-stage exchange for PJI

The OPTION trial is a multicenter, prospective, randomized clinical trial comparing one-stage versus two-stage exchange arthroplasty for chronic PJI (PJI with a symptom duration of more than 4 weeks after the index arthroplasty or a late infection occurring more than 4 weeks postoperatively).

Led by researchers at OrthoCarolina Research Institute, the trial enrolled 321 patients with chronic PJI with a known organism of primary hip and knee arthroplasties, as defined by Musculoskeletal Infection Society (MSIS) criteria (15). Patients were randomized to receive either one-stage (164 patients) or two-stage (157 patients) exchange. The study excluded patients with revision arthroplasties, fungal infections, immunosuppression, or compromised soft tissue precluding wound closure. All participants, regardless of treatment arm, underwent standardized protocols, similar irrigation procedures, 6 weeks of intravenous antibiotics initially, and 6 months of oral antibiotics following reimplantation (16).

The primary outcome measure was treatment success at 1 year, defined as the absence of reoperation for PJI. In the interim analysis, 245 patients (128 one-stage and 117 two-stage) had completed 1-year follow-up, while 50 of 321 patients (16%) were lost to follow-up, and 26 patients (8%) had not yet completed the study (17). The preliminary results showed a success rate of 125/128 (98%) for one-stage exchange compared to 110/117 (94%) for two-stage exchange, with no statistically significant difference between groups (*P* = 0.15). The one-stage group demonstrated reduced relative risk of failure (RR 0.39; 95% CI 0.10–1.4) compared to two-stage exchange, though this difference was not statistically significant after adjusting for age and MSIS host classification 1.02 (95% CI 0.99–1.04). Adverse event rates were comparable between groups (32% one-stage vs. 38% two-stage, *P* = 0.27) (17).

Patient enrollment commenced in January 2021, with anticipated completion of 2-year follow-up data collection by December 2025. These interim results add to emerging data that challenge the current consensus favoring two-stage exchange arthroplasty for chronic PJI (24). The preliminary findings suggest potential advantages of single-stage procedures in appropriately selected patients, with implications for both clinical outcomes and resource utilization. However, definitive conclusions regarding the optimal surgical approach await completion of the pre-specified 2-year follow-up period.

A significant limitation of this study lies in its definition of treatment success, which is solely based on the absence of reoperation for PJI. This definition may overestimate success rates, as it fails to capture important clinical scenarios such as patients on chronic suppressive antibiotics who would be classified as “successes” despite not achieving true infection resolution. This limitation should be considered when interpreting the success rates, which appear notably higher than those previously reported in the literature (25).

### CORGI trial: controlled trial of omadacycline for bone and joint infection

Omadacycline is a novel aminomethylcycline antibiotic with broad-spectrum activity against gram-positive bacteria, including strains resistant to earlier generation tetracyclines, as well as certain gram-negative and atypical pathogens. CORGI is a prospective, open-label RCT comparing omadacycline with standard-of-care (SOC) oral antibiotics for the treatment of bone and joint infections (BJI), including PJI. Led by researchers at the Lundquist Institute for Biomedical Innovation at Harbor-UCLA Medical Centre, the trial aims to enrol 180 patients with BJI requiring 4–12 weeks of antibiotic therapy. Patients are randomized to receive either omadacycline 300 mg once daily orally or SOC oral antibiotics as determined by their treating physician prior to enrollment (18).

The study excludes PJI that have not undergone both stages of two-stage surgical treatment, meaning patients are only eligible after the second-stage surgery has been completed and typically 6 weeks of intravenous therapy has been administered. This would target only a specific PJI population: those in the post-reimplantation phase requiring prolonged oral antibiotic therapy for infection consolidation. The study also excludes patients with fungal or mycobacterial infections, BJI complicated by endocarditis or central nervous system involvement, and hematogenous BJI prior to adequate bacteremia treatment. 

The primary outcome measure is treatment success at 2 weeks post-therapy completion, defined as the absence of definite treatment failure using criteria adapted from the OVIVA trial. Treatment failure is defined by clinical criteria (draining sinus tract arising from bone or prosthesis, or frank pus adjacent to bone or prosthesis), microbiologic criteria (phenotypically indistinguishable bacteria from two or more deep-tissue samples, pathogenic organism from single closed aspirate or biopsy of peri-prosthetic tissue), or histologic criteria (characteristic inflammatory infiltrate or microorganisms). For PJI patients specifically, additional failure criteria include peri-prosthetic necrotic bone and rapid loosening of a joint prosthesis leading to localized pain within 3 months of implantation without mechanical explanation. Secondary endpoints include long-term treatment success at 3 months post-therapy completion, safety and tolerability, and medication adherence measured by self-report.

Patient enrollment commenced in May 2023, with primary completion estimated for December 2025. As of October 2024, interim safety data from 59 enrolled participants have been reported. Of these patients, 31 (53%) were randomized to the omadacycline-containing regimen and 28 (47%) to SOC. The interim analysis revealed that diabetic foot infections comprised the vast majority of cases (52 patients, 88%), while PJI represented 3 patients (5%) of the cohort to date (19). This distribution highlights a critical limitation for interpreting PJI-specific outcomes, as the overwhelming predominance of diabetic foot infections may limit the generalizability of results to PJI management. Furthermore, within the small PJI subset, the types of prostheses involved (hip versus knee, primary versus revision components), the complexity of the surgical procedures, and the causative organisms will be important determinants of treatment success.

Several other limitations warrant consideration. The short-term primary outcome assessment at 2 weeks post-treatment completion is inadequate for PJI evaluation, given that treatment failures frequently manifest months or even years after therapy completion. The open-label design may introduce bias in outcome assessment, particularly given the subjective nature of some clinical endpoints. For PJI patients specifically, the exclusion of those requiring indefinite suppressive therapy narrows the applicable population, as chronic suppression represents a significant management strategy for certain PJI cases. Furthermore, by limiting enrollment to post-reimplantation PJI patients who have completed two-stage procedures, the study excludes other important PJI management scenarios, such as one-stage revisions or acute infections managed with debridement, DAIR (19).

### Can “Lite” procedure combined with a short course antibiotic treatment be effective in treating chronic PJI?

The Long Interval Two-stage Exchange (LITE) trial represents a prospective RCT investigating the optimal duration of antibiotic therapy following two-stage exchange arthroplasty for chronic PJI. Led by researchers at the First Affiliated Hospital of Fujian Medical University in China, the study enrolled 60 patients with chronic PJI who completed the LITE procedure between April 2018 and November 2021. Following completion of the second stage of reimplantation, patients were randomized to receive either a short course (2 weeks) or a long course (12 weeks) of postoperative antibiotics. The study included patients diagnosed with PJI according to MSIS criteria and classified as Tsukayama type III (late chronic infection occurring 4 weeks post-primary arthroplasty). Exclusion criteria encompassed patients with fungal, mycobacterial, or tuberculosis infections, unclear pathogens during first-stage surgery, positive cultures during the interval period, and those who did not pass infection control assessment during the interval (20).

The LITE procedure involved a standardized protocol with two distinct surgical stages separated by a prolonged interval of at least 12 weeks. The first stage consisted of prosthesis removal, comprehensive debridement, and antibiotic-impregnated bone cement spacer implantation. This was followed by at least 6 weeks of intravenous and/or oral antibiotics based on culture results and microbiologist guidance, succeeded by a minimum of 6 weeks of antibiotic holiday period for infection monitoring. Second-stage reimplantation was performed 3 months after the first-stage surgery, provided inflammatory markers had significantly decreased and remained stable during the holiday period (20).

The primary outcome measure was infection control rate, defined as the absence of clinical signs and symptoms, laboratory and imaging indications, or any other evidence of infection following the LITE procedure and subsequent antibiotic therapy, with no continuous requirement for antibiotic use to suppress infection at 24 months. Secondary outcomes included antibiotic-related complications, cost of antibiotic treatment, and long-term functional outcomes. All patients were followed for a minimum of 24 months, with a mean follow-up of 39.2 ± 13.0 months. The study was powered to demonstrate non-inferiority of 2 weeks of antibiotic regimen to 12 weeks of regimen using a non-inferiority margin of 15%.

The study population was well-balanced between groups, with 30 patients randomized to each arm. The cohort was predominantly female (63.3%) with a mean age of approximately 62 years. Hip PJI was more common than knee PJI (55% versus 45%), and patients had similar comorbidity profiles, including diabetes (26.7%), with no significant differences in baseline inflammatory markers between groups. The most common causative organisms were *S. aureus* (35%), coagulase-negative staphylococci (28.4%), and gram-negative bacilli (18.3%). Eight patients had methicillin-resistant *S. aureus* (MRSA) infections (13.3%), and nine had methicillin-resistant *S. epidermidis* (MRSE) infections (15.0%), with no significant difference in pathogen distribution between groups.

The primary findings demonstrated that infection control rates were identical between the short-course and long-course groups (96.7% versus 96.7%, *P* = 1.000). In the short course group, one patient with MRSE infection developed uncontrolled infection requiring extended oral linezolid therapy but ultimately achieved infection control without reoperation. In the long-course group, one patient developed reinfection with *Stenotrophomonas maltophilia* 18 months post-reimplantation, which was successfully managed with DAIR (20).

The study revealed significant differences in safety and economic outcomes favoring the short course approach. No patients in the short course group developed antibiotic-related complications, while six patients (20%) in the long course group experienced complications, including abnormal liver function (five cases) and hematopoietic dysfunction (one case) (*P* = 0.024). All complications resolved within 3 months after antibiotic discontinuation. Furthermore, the cost of antibiotic treatment post-LITE procedure was significantly lower in the short course group compared to the long course group (9,004.3 ± 6,980 Chinese Yuan [Chinese Yuan] versus 24,585.5 ± 22,509 CNY, *P* = 0.001), representing a substantial reduction in treatment costs (20).

The study’s high infection control rate of 96.7% was attributed to several factors: enhanced pathogen detection during first-stage surgery through combined culture and next-generation sequencing analysis of synovial fluid, sonicate fluid, and tissue samples; administration of antibiotic therapy for at least 6 weeks following first-stage surgery; and implementation of a minimum 6 week antibiotic holiday to monitor infection control. These measures facilitated the identification of patients with uncontrolled infections, with two patients excluded due to the detection of the same pathogens during the interval period.

A notable strength of this study is its focus on a clinically relevant question regarding antibiotic duration optimization following two-stage exchange. The standardized LITE protocol with prolonged interval and comprehensive infection monitoring provides a robust framework for evaluating shortened antibiotic courses. The study’s randomized design and comprehensive follow-up enhance the validity of its findings.

However, several limitations warrant consideration. The single-center design with treatment by the same medical team, while ensuring protocol consistency, may limit the generalizability of results. The exclusion of patients with positive cultures during the interval and second-stage reimplantation, though clinically appropriate, potentially contributed to the observed high infection control rates and may not reflect real-world scenarios where such complications occur. In addition, the exclusion of fungal and mycobacterial infections limits the applicability of findings to these challenging pathogen groups. The study’s focus on patients who successfully completed the LITE procedure excludes consideration of those who failed the first stage, representing a selected population with potentially better prognosis.

The findings have important implications for PJI management, suggesting that 2 weeks of course of postoperative antibiotics following successful two-stage exchange may be sufficient for infection control while reducing complications and costs. However, even 2 weeks of antibiotics might not be needed after the second stage, and the ROADMAP trial will be able to answer this question.

## INVESTIGATIONAL PRODUCTS

### VT-X7: local vancomycin-tobramycin combination for two-stage exchange

VT-X7, developed by Osteal Therapeutics, is a novel drug-device combination designed for two-stage revision arthroplasties. The system delivers high concentrations of vancomycin and tobramycin directly to the infected joint space through cyclical irrigation and evacuation. After the removal of the infected prosthesis in the first stage, the VT-X7 system is placed in the joint space for a 7-day treatment period, during which it continuously flushes the area with antibiotics. The system is removed prior to the second-stage reimplantation of the new prosthesis. This approach aims to not only provide targeted local antibiotic delivery but also potentially reduce the interstage period, which traditionally ranges from 6 to 12 weeks. (Table 2).

**TABLE 2:** Industry-sponsored trials in PJI management^*a*^

Trial name	Phase	Surgical strategy	Intervention	Status	Key outcome measure	Key results	Reference
VT-X7 APEX	1	Two-stage revision	Novel vancomycin-tobramycin delivery system vs standard antibiotic-loaded cement spacers	Completed	Safety and efficacy in two-stage exchange	Demonstrated safety	(26–28)
VT-X7 APEX-2	2	Two-stage revision	Vancomycin-tobramycin delivery system	Recruiting (began early 2023)	Safety and treatment efficacy compared to the SoC	Demonstrated safety; three device-related complications and three cases of acute kidney injury in the experimental group. Treatment efficacy not available.	(27)
PLG0206	1b	DAIR only	Engineered antibacterial peptide as surgical irrigation in addition to systemic antimicrobials	Completed	Safety and tolerability	93% (13/14) infection-free at 180 days; no treatment-related serious adverse events	(29, 30)
TRL1068 Phase 1	1	Two-stage revision	Monoclonal antibody for biofilm distruption in addition to SoC antimicrobials	Completed	Safety and bacterial elimination	Complete bacterial elimination in 3/11 treated patients; all remained infection-free through 169 days	(31)
TRL1068 Phase 2	2	DAIR only	Monoclonal antibody for biofilm distruption with the DAIR procedure	Site initiation to begin in May 2025	Efficacy and safety with the DAIR procedure	Not yet available	(32)
TNP-2092 Tissue Distribution	1	Primary arthroplasty	Tissue distribution and pharmacokinetics in primary arthroplasty	Completed	Bone and synovial fluid concentrations	Achieved concentrations exceeding the minimum biofilm bactericidal concentration for *S. epidermidis*	(33, 34)
TNP-2092 Cohort study	1	Not applicable	Dose escalation study in healthy Chinese participants	Completed	Safety and pharmacokinetics	Safe and well-tolerated up to 400 mg IV; comparable pharmacokinetics to the US population	(35, 36)
Afabicin Phase 2	2	Not specified (bone and joint infections)	Afabicin vs standard of care for staphylococcal bone and joint infections	Ongoing	Safety, tolerability, and efficacy	Preliminary: Safety demonstrated. All 20 patients (17 afabicin, 3 SoC) were treatment responders at the end of the treatment	(37)
PhagoDAIRI	2	DAIR only	Anti-Staphylococcal bacteriophages for *S. aureus* PJI with DAIR	Study status uncertain due to Phaxiam bankruptcy	Clinical control of infection at week 12	80% infection control rate in the first 20/64 patients	(38)
GLORIA	2	DAIR only	Anti-Staphylococcal bacteriophages for *S. aureus* PJI with DAIR	Study status uncertain due to Phaxiam bankruptcy	Clinical cure and safety up to 3 months	Not yet available	(39)

^*a*^ DAIR = debridement, antibiotics, and implant retention; THA = total hip arthroplasty; TKA = total knee arthroplasty; PJI = prosthetic joint infection; SoC = standard of care.

The initial feasibility study (NCT03721328) focused on the safety assessment of VT-X7 where it was found to be safe (26). The APEX trial (NCT04662632) was designed as a Phase 2 multicenter randomized controlled trial to evaluate VT-X7 in two-stage exchange arthroplasty. The study was conducted across 17 US centers between November 2021 and November 2022, enrolling 76 patients who were randomized to either experimental (*n* = 37) or control (*n* = 39) groups (27). The sample size calculation was based on assumptions of a 50% success rate for two-stage exchange arthroplasty (notably low) and powered to detect a 35% difference (notably high) in success at 90 days post first stage with 90% power.

The experimental protocol involved a first-stage resection arthroplasty followed by placement of the VT-X7 system. During the 7-day interstage period, patients received daily intra-articular doses of tobramycin (80 mg in 50 mL saline) with 2 hours of soak and 30 minutes of vacuum drainage, followed by hourly intra-articular irrigation with vancomycin (125 mg in 50 mL saline) with 30 minutes of soak and vacuum cycles. This regimen delivered 22 daily cycles, totaling 2,750 mg vancomycin and 80 mg tobramycin locally. Concurrent systemic antibiotics were permitted, though systemic vancomycin and tobramycin were discouraged during this period. The control group underwent standard treatment with antibiotic-loaded cement spacers containing an average of 16 g total antibiotics (8.4 g vancomycin, 7.1 g tobramycin), concurrent with a minimum of 6 weeks of organism-specific IV or oral antibiotics and 2 weeks of antibiotic holiday before the second stage. Both groups received 12 weeks of systemic antibiotics following second stage (28). Pharmacokinetic analysis revealed detectable serum levels in 69% of vancomycin and 45% of tobramycin measurements in the experimental arm, though most remained below 1 mg/mL, and all were below 2 mg/mL in peak testing. Two notable exceptions occurred when patients received additional IV vancomycin against protocol recommendations, resulting in serum vancomycin concentrations of 36.2 and 35.1 mg/mL (the manuscript does not specify whether these were trough levels or measured at another time point). While adverse event rates were generally similar between groups, the experimental group experienced three device-related complications involving installation line breakage and retained broken drains. In addition, three cases of acute kidney injury occurred in the experimental group within 180 days post-first stage, with two classified as serious adverse events. One case was attributed to excessive systemic vancomycin use, while another occurred 4 days adter second stage in a patient receiving concurrent antibiotics. No kidney injuries were reported in the control group (28).

These findings led to multiple FDA designations for VT-X7, including Orphan Drug, Fast Track, Qualified Infectious Disease Product, and Breakthrough Therapy status (40). However, several important considerations emerged from the study. The complexity of the VT-X7 delivery system compared to conventional antibiotic-loaded cement spacers or biodegradable components requires careful evaluation. Recent findings from the SOLARIO trial suggest that local antibiotics delivered via cement spacers or biodegradable components might be sufficient with just 7 days of systemic antibiotics post-first stage. Furthermore, the potential side effects and complications associated with the delivery system, along with the need for larger studies to demonstrate superiority over current standards of care, underscore the importance of continued investigation. A follow-up APEX-2 Phase II trial (NCT05607030) began enrollment in early 2023 to further evaluate the treatment efficacy of VT-X7 compared to standard of care.

### PLG0206: engineered antibacterial peptide

PLG0206 (previously WLBU2) is an engineered cationic amphipathic peptide (CAP). The compound was designed with an idealized helical structure to optimize bacterial membrane selectivity while minimizing host cytotoxicity. By altering bacterial membrane permeability, PLG0206 exhibits broad-spectrum activity against both Gram-positive (*Staphylococci, Enterococci*) and Gram-negative bacteria (*Escherichia coli*, *Klebsiella pneumoniae*, *Enterobacter cloacae*, *Pseudomonas aeruginosa*, *Acinetobacter baumannii*), maintaining effectiveness regardless of antibiotic resistance profiles (41, 42). While PLG0206 demonstrates advances over traditional CAPs, its clinical development is most justified in cases where conventional antibiotics have failed (41). As such, more recent studies have focused on its potential in preventing and disrupting bacterial biofilms, for example, against *P. aeruginosa* biofilms associated with cystic fibrosis (43) and against MSSA/MRSA surgical implant infection (41).

Huang et al. investigated its effectiveness in treating chronic PJI using explanted knee implants from 17 patients (42). The experimental protocol involved treating explanted prosthetics with PLG0206 at a 1 mg/mL concentration for 15 minutes, a parameter established through previous *in vitro* time-kill studies and animal models. The explants showed diverse bacterial colonization, with *Staphylococcus epidermidis* (35%), *S. aureus* (18%), and *E. coli* (12%) being the predominant species. Treatment with PLG0206 resulted in complete bacterial eradication in 59% of samples and achieved a mean 4-log10 reduction in bacterial burden (range 1–7 logs), supporting PLG0206’s potential as 15 minutes of local irrigation solution at 1 mg/mL concentration for PJI treatment in knee and hip arthroplasties.

The Phase 1b open-label, dose-escalating trial (NCT05137314) evaluated PLG0206 irrigation in patients undergoing DAIR following total knee arthroplasty (29). The study enrolled 14 patients across multiple US centers. The trial design incorporated two seven-patient cohorts receiving single-dose escalating treatments of either 3 mg/mL or 10 mg/mL PLG0206 delivered as surgical irrigation in addition to the 6 weeks of course of systemic antimicrobial therapy. The results are compared to historical control patients who received the current standard of care, consisting of saline and betadine irrigation during DAIR surgery (44). Interim data showed infection-free status in 13 of 14 patients (93%) at 180-day follow-up, with no treatment-related serious adverse events reported (30). However, these results require cautious interpretation given the small sample size, lack of concurrent controls, short follow-up period, and early-stage nature of the trial. While these early results suggest PLG0206 could potentially enhance the treatment options for PJI, larger cohort studies with extended follow-up periods will be necessary to fully establish its efficacy in addition to systemic antimicrobial therapy and long-term safety profile.

### TRL1068 (calpurbatug): monoclonal antibody for biofilm disruption

TRL1068 (calpurbatug) is a native human monoclonal antibody targeting a highly conserved epitope on DNABII proteins, a family of bacterial proteins that provide structural cohesion for biofilms of both Gram-positive and Gram-negative bacteria (45). With high binding affinity (Kd ~50 pM), TRL1068 extracts these scaffolding proteins that hold in place bacterial-derived extracellular DNA (eDNA), which constitutes up to 50% of biofilm mass (46). The epitope is conserved across hundreds of bacterial species. This conservation explains TRL1068’s *in vitro* activity against major biofilm-associated pathogens such as *S. aureus*, *P. aeruginosa*, *A. baumannii*, *K. pneumoniae*, *Streptococcus pneumoniae*, *Haemophilus influenzae*, *Borrelia burgdorferi*, and *Enterococcus* species.

Burke et al. demonstrated TRL1068’s preclinical efficacy using a mouse MSSA spinal pin infection model (47). The study design involved implanting stainless steel pins into the L4 spinous process, with mice divided into four groups: sterile control, infected control, vancomycin monotherapy, and TRL1068 + vancomycin combination therapy. Following *S. aureus* inoculation on day 0, a 4-day period allowed biofilm establishment before initiating treatment. The treatment protocol consisted of systemic TRL1068 (15 mg/kg subcutaneously) administered on days 4 and 7, combined with vancomycin (120 mg/kg every 12 hours) from days 7 to 21. A further 14 days without treatment was included in the study design to allow the biofilm to reform. At day 35, analysis revealed MSSA recovery in only 3.7% (1/27) of TRL1068 + vancomycin-treated animals, compared to 41.7% (5/12) in the vancomycin monotherapy group and 66.7% (8/12) in infected controls. This represented a 97% reduction in bacterial load compared to controls, achieved through systemic treatment without surgical debridement. Electron microscopy provided the first *in vivo* evidence of TRL1068-mediated biofilm disruption (47).

Building on these preclinical findings, a Phase 1 clinical trial evaluated TRL1068 pharmacokinetics and pharmacodynamic characteristics in chronic PJI patients undergoing two-stage exchange arthroplasty of the knee or hip (31). The study enrolled 15 patients (11 TRL1068, 4 placebo), with TRL1068 administered as a single 60 minute intravenous infusion on day 1 in three ascending dose groups (6, 15, and 30 mg/kg). All patients received pathogen-directed antibiotics until removal of the infected prosthesis on day 8, followed by antibiotic-eluting spacer placement and 6 weeks of parenteral antibiotics as per standard of care before implanting a new prosthetic joint. Preoperative synovial fluid cultures confirmed diverse bacterial infections, including mono- and polymicrobial cases with both Gram-positive and Gram-negative species. These included Staphylococcus species, *P. aeruginosa*, *E. cloacae*, Streptococcus species, and various others. Three out of 11 TRL1068 treated had no detectable bacterial pathogens in cultures of sonicated explanted prostheses at day 8 despite initial positive cultures. Among the three patients who achieved complete bacterial elimination within 8 days, Patient 4 with *P. aeruginosa* had 1 CFU/mL, which is considered below the threshold of infection for the sonication method used. Patient 11 had an initial *Streptococcus agalactiae* infection and showed no infection of the sonicate, and Patient 12 presented with both *P. aeruginosa* and Klebsiella sp. and also showed no infection of the sonicate. Three additional polymicrobial patients had elimination of one bacterial species of multiple bacterial species present at Baseline. Patient 5 initially presented with *Enterobacter cloacae* and *S. maltophilia*, and while *Enterobacter* was eliminated, *Stenotrophomonas* was not. The authors noted that the additional bacterial species found in the sonication culture were likely surface colonizers due to wound dehiscence. Patient 10 had an initial *Streptococcus pneumoniae* and *Enterococcus faecalis* infection, and the Streptococcus infection was eliminated. Patient 13 had an initial *Streptococcus mitis* and *Staphylococcus lugdunensis* infection, and the Streptococcus infection was eliminated. The remaining five TRL1068-treated patients showed varying degrees of residual bacteria. Two patients with monomicrobial *S. lugdunensis* infections demonstrated different responses: one showed a low bacterial count (13 CFU/mL), while the other had >100 CFU/mL on Day 8. A patient with *Cutibacterium acnes* showed minimal persistence (10 CFU/mL). Two patients with coagulase-negative Staphylococcus showed different outcomes: one with 50 CFU/mL and another with >100 CFU/mL of *S. lugdunensis*. Authors compared the findings to historical controls from the Trampuz et al. study, which established the sonication procedure for measuring the bacterial burden on explanted prostheses; in these historical controls, 85% (51/60) of explanted prostheses (without antibiotic pretreatment) showed ≥100 CFU/mL bacterial growth, in contrast to only 36% (4/11) of TRL1068-treated patients reaching this threshold (48). In addition, despite varying levels of bacterial persistence, all 11 TRL1068-treated patients remained free from recurrent infection with their initial pathogens through the 169-day observation period.

The authors attributed the reduced efficacy against Staphylococcus to these species having less extracellular DNA, and thus lower concentrations of DNABII-binding proteins in their biofilms, relying instead on protein aggregates like fibrinogen and albumin to provide structural coherence. The authors also noted that the 7-day treatment period might have been insufficient for maximum antimicrobial activity. This observation was supported by preclinical mouse data showing that full penetration of TRL1068 into subcutaneous implants required at least 3 days, suggesting potentially longer distribution times in human bone-implant interfaces (46).

The development program has now advanced to Phase 2 (NCT06621251), with FDA agreement on the trial design. This upcoming study, which has begun site initiation in May 2025, aims to evaluate the efficacy and safety of TRL1068 when administered in conjunction with a DAIR procedure (32). If successful, TRL1068 could offer a less invasive alternative to current standard-of-care treatments by preventing chronic PJI cases from requiring staged revision arthroplasty.

### TNP-2092 (rifaquizinone): dual-action antibacterial agent

TNP-2092 is a semi-synthetic hybrid compound that combines rifamycin and quinolone pharmacophores. This design follows the established strategy of developing dual-acting antibacterial molecules that target bacteria through two distinct mechanisms of action. The aims are to overcome rifampin resistance development and leverage the antibiofilm activity of rifampin in combination with ciprofloxacin against staphylococcal species. TNP-2092 inhibits three bacterial targets simultaneously: DNA gyrase, topoisomerase IV, and RNA polymerase (49).

The compound demonstrated potent activity against PJI-associated staphylococci with MICs ≤ 0.125 µg/mL, for all strains tested, including MRSA and MSSE, and also those resistant to both rifampin and ciprofloxacin (50). It has also demonstrated *in vitro* activity against various bacterial species, including, methicillin-resistant *S. epidermidis* (MRSE), methicillin-susceptible *S. epidermidis* (MSSE), streptococci, and *Helicobacter pylori* (51).

*In vivo* efficacy of intraarticular TNP-2092 was assessed in a rat PJI model with MRSA and MSSA (52). Rats were randomized to receive either daily intraarticular TNP-2092 at different doses for 14 days or intraarticular vancomycin. Both high-dose TNP-2092 and vancomycin groups showed significant improvement in clinical signs of PJI (reduced knee swelling and temperature), local and systemic inflammatory markers, and microbiological load compared to untreated controls, with comparable efficacy between the two treatment groups. Electron microscopy demonstrated superior anti-biofilm activity of TNP-2092 compared to vancomycin.

A Phase 1 mass balance study using [14C]-labeled TNP-2092 showed that after intravenous administration, the compound was primarily eliminated in feces (98.7%) with minimal urinary excretion (2.7%). The drug showed good distribution characteristics with a plasma half-life of approximately 0.77 hours (53).

Another Phase 1 trial (NCT04294862) evaluated TNP-2092’s tissue distribution and pharmacokinetics in 13 primary arthroplasty patients (9 hip and 4 knee). The study protocol involved administering a single 300 mg intravenous dose of TNP-2092 two hours before anesthesia induction, with bone and synovial fluid samples collected during surgery (33). The results showed that after a single 300 mg intravenous dose, TNP-2092 achieved concentrations in bone and synovial fluid that exceeded the minimum biofilm bactericidal concentration for *S. epidermidis* in both total hip and knee arthroplasty patients. In hip arthroplasty patients, concentrations also exceeded the minimum biofilm bactericidal concentration for *S. aureus* in the acetabulum and synovial fluid (34).

In parallel, a Phase 1 dose escalation study in healthy Chinese participants examined three dosing regimens: 200 mg single dose, 300 mg multiple dose (with additional doses every 12 hours from days 4 to 11), and 400 mg single dose (35). It was found to be safe and well-tolerated at doses up to 400 mg when administered intravenously. The pharmacokinetics were comparable between Chinese and US populations, with slightly slower clearance (13.9%) in Chinese participants (36). The complete results, including synovial and blood level measurements, are pending publication.

A subsequent Phase 2 trial of rifaquizinone enrolled patients with acute bacterial skin and skin structure infections (NCT03964493) (54). This multicenter, double-blind, randomized, active-controlled study compared rifaquizinone with vancomycin in 120 adult patients requiring intravenous antibiotic treatment for infections suspected or confirmed to be caused by Gram-positive pathogens (MRSA 62.7% rifaquizinone group vs 65.5% vancomycin group). Patients received either rifaquizinone 300 mg (*n* = 80) or vancomycin 1 g (*n* = 40) intravenously every 12 hours for 3–14 days. The primary endpoint was early assessment response, defined as survival with at least a 20% reduction in lesion size without rescue antibiotics. Rifaquizinone demonstrated numerically higher response rates across all populations, with 78.1% response in MRSA infections compared to 57.9% with vancomycin. The safety profile was comparable between treatments, with serious adverse events rare in both groups (1.3% rifaquizinone vs 5.1% vancomycin) (54).

According to information from clinicaltrials.gov and TenNor Therapeutics’ website, following successful safety demonstrations in these initial studies, TNP-2092 has progressed to multiregional clinical trials for both PJI and skin and soft tissue infections. These trials have received regulatory approval from both the US FDA and the China National Medical Products Administration (55).

While TNP-2092 appears to be a promising addition to the antimicrobial armamentarium for PJI treatment, more robust clinical evidence is needed before its place in therapy can be established.

### Afabicin (Debio 1450): FabI inhibitor for Staphylococcal infections

Afabicin (Debio 1450) is a first-in-class antibiotic that operates through a unique mechanism of action, selectively targeting the FabI enzyme essential for bacterial fatty acid synthesis in Staphylococcus species (56). Similar to TNP-2092 (Rifaquizinone), this semi-synthetic prodrug combines two distinct pharmacophores: a natural rifamycin component with a synthetic quinolizinone (a quinolone isomer). While the prodrug itself has no antimicrobial activity, its active moiety, afabicin desphosphono (Debio 1452), has demonstrated potent activity against both coagulase-positive and coagulase-negative staphylococcal strains, including the methicillin-resistant strains (57).

*In vitro* analysis of 872 isolates showed afabicin MIC ranges of 0.002–0.25 µg/mL and MIC90 values of 0.016 µg/mL against clinical *S. aureus* isolates (58). Animal studies established pharmacodynamic targets relevant to bone and joint infections, and clinical pharmacokinetic analyses confirmed that selected dosing regimens consistently achieved target exposures (58).

A multicenter Phase 2 trial evaluated oral afabicin compared with IV vancomycin/oral linezolid for staphylococcal skin and soft tissue infections (57). A total of 330 patients were randomized to receive low-dose afabicin (80 mg IV → 120 mg oral twice daily), high-dose afabicin (160 mg IV → 240 mg oral twice daily), or vancomycin/linezolid. The primary endpoint was early clinical response (≥20% lesion reduction) at 48–72 hours. Both afabicin doses demonstrated non-inferiority to vancomycin/linezolid for the primary endpoint (94.6% and 90.1% vs 91.1%, respectively). However, at short-term follow-up (14 days post-treatment), clinical success rates were lower with afabicin (84.8% and 83.5%) compared to vancomycin/linezolid (92.1%), suggesting potential concerns about durability of response that may be particularly relevant for PJI treatment (57).

An ongoing Phase 2 trial (NCT03723551) investigates the safety, tolerability, and efficacy of afabicin compared to standard of care (SoC) in patients with bone and joint infections (BJI) caused by MSSA, MRSA, or CoNS. Preliminary results from this study were presented at IDweek 2024 (37). The trial evaluated a dosing regimen of 55 mg IV BID followed by 80 mg oral BID for 2–3 weeks. Among the 20 patients in the microbiological intent-to-treat population (17 afabicin, 3 standard of care), osteomyelitis was the predominant diagnosis (82.4% in the afabicin group, 100% in the SoC group) (37). The study reported that all 20 patients in the microbiological intent-to-treat (mITT) population (17 afabicin and 3 SoC) were treatment responders at the end of treatment (EoT). Clinical response at 4 weeks post-EoT was 13 out of 15 patients in the afabicin arm and all 3 patients in the SoC arm. Clinical response at 12 weeks post-EoT was 13 out of 15 patients in the afabicin arm and 2 out of 3 patients in the SoC arm. These results suggest that afabicin has comparable efficacy to SoC in treating staphylococcal BJI. The study also noted that afabicin had a similar safety profile to the SoC arm. While the study presents promising results for afabicin, several limitations should be considered: small sample size; lack of surgical information; and short follow-up period. Despite these limitations, the study provides preliminary evidence suggesting that afabicin could be a safe and effective treatment option for staphylococcal BJI.

### Phage therapy for PJI

Two ongoing clinical trials by Phaxiam Therapeutics were investigating bacteriophage therapy as a treatment for *S. aureus* PJI in the hip and knee, though the company’s recent financial collapse has placed these studies in significant uncertainty (59).

The PhagoDAIRI trial (NCT05369104), which began in June 2022, is a pilot, multicenter, randomized, double-blind study evaluating phage therapy in patients with *S. aureus* PJI treated with the DAIR procedure and suppressive antibiotic therapy. The study includes patients with *S. aureus* monomicrobial knee or hip PJI occurring more than 3 months after prosthesis implantation who present with clinical signs of infection and have an indication for DAIR with direct closure and suppressive antibiotic therapy. Early *S. aureus* prosthetic joint infections (less than 3 months after prosthesis implantation) are excluded (38). This Phase II study has an estimated enrollment of 64 participants and is being conducted in Lyon, France. Participants are randomized to receive either anti-Staphylococcal bacteriophages (PP1493, PP1815, or both) or placebo (NaCl 0.9% solution) as a single intra-articular injection at the end of the DAIR procedure in addition to the suppressive antibiotic therapy (38). The primary outcome measure is clinical control of infection (defined as no fever, no recurrence, no worsening of pain, no periarticular inflammatory and clinical infectious signs, no new surgical request, and no *S. aureus* detected in joint fluid) at the week 12 visit. Secondary endpoints include clinical control of infection at 6, 12, 18, and 24 months.

Building on this work, the GLORIA study (NCT06605651) was planned as a more extensive Phase II proof-of-concept trial scheduled to begin early 2025. This international, multicenter, randomized, double-blind, placebo-controlled study aimed to assess the safety and efficacy of phage therapy versus placebo in treating *S. aureus* infections in hip or knee PJI patients undergoing DAIR (39). With an estimated enrollment of 100 participants, this study employed a more intensive treatment protocol, featuring three intra-articular injections of either anti-*S*. *aureus* bacteriophages (PP1493 and PP1815) or placebo during and/or following the DAIR procedure (39). Inclusion criteria included indication for open DAIR as decided by the multidisciplinary team and/or principal investigator, while indication for suppressive antibiotic therapy was an exclusion criterion. This was a quadruple-blind study with participant, care provider, investigator, and outcomes assessor all blinded to the treatment arm. The primary outcome measure was to assess efficacy (percentage of patients with clinical cure) and safety (incidence of serious adverse events) of phage therapy + DAIR compared to placebo from enrollment up to 3 months, with secondary outcomes including clinical cure at 6 months and up to 12 months. At present, clinicaltrials.gov does not list the participating research centers for this study (39).

According to Phaxiam’s website, the company had treated more than 120 patients under compassionate use protocols since 2017, primarily for hip or knee PJI among other bone and joint infections (60, 61). Data released in January 2025 from 88 evaluated patients showed an infection control rate of 75% at 3 months post-treatment. While the definition of infection control is not explicitly stated on the website, it was defined in the clinicaltrials.gov pages for the PhagoDAIRI and GLORIA studies as no fever, no recurrence, no worsening of pain, no periarticular inflammatory and clinical infectious signs, no new surgical request, and no *S. aureus* detected in joint fluid. Of particular note are the 52 patients with PJI caused by either *S. aureus* (*n* = 40) or *P. aeruginosa* (*n* = 12), who reportedly achieved 79% infection control at 3 months (77% for *S. aureus* and 83% for *P. aeruginosa*). At 12 months, Phaxiam reports infection control rates between 64% and 67%, though it should be noted that these data are incomplete. Phaxiam also claims these results are consistent with their PhagoDAIRI pilot study, which showed an 80% (16/20) infection control rate in 20 patients out of 64 that the study plans to enrol. However, it is important to recognize that these are company-reported outcomes without peer-reviewed publication. According to a review article by Suh et al., who examined all bone and joint infection cases reported in the literature that were treated with phages, of 33 reported cases treated with phage therapy, 29 (87%) achieved microbiological or clinical success, 2 (5.9%) relapsed with the same organisms, and 2 (5.9%) with a different organism. Of these four relapses, all but one eventually achieved clinical resolution with additional surgery or phage treatments (62).

However, in May 2025, Phaxiam Therapeutics announced that the Lyon Commercial Court had initiated judicial liquidation proceedings, effectively declaring the company bankrupt. The company warned that shareholders would likely receive no reimbursement due to its level of indebtedness, and shares will be delisted from Euronext. This financial collapse places the future of both the PhagoDAIRI and GLORIA trials in serious doubt, representing a significant setback for bacteriophage therapy development in PJI treatment (59).

These trials and compassionate use programs represented important steps in evaluating bacteriophage therapy for orthopedic infections, but the company’s bankruptcy highlights the significant clinical and commercial challenges that remain before phage therapy can be considered a mainstream treatment option for PJI.

## TRIALS ON PJI PREVENTION

### ProHipQ-OA: single versus multiple dose antibiotic prophylaxis in primary THA

The Pro-Hip-Quality OA trial (NCT05530551), conducted in Denmark, is designed to include approximately 20,000 patients across 36 hospitals (21). This trial focuses on patients undergoing primary total hip arthroplasty (THA) due to primary and secondary osteoarthritis, excluding those with THA due to fractures, tumors, or metastasis. The trial aims to determine whether a single preoperative intravenous dose of dicloxacillin or cefuroxime is as effective as multiple doses in preventing PJI within 90 days after surgery.

The single-dose regimen involves administering dicloxacillin (2 g for those under 120 kg, 3 g for those 120 kg and above) or cefuroxime (1.5 g for those under 120 kg, 3 g for those 120 kg and above) within an hour before the incision. The multiple-dose group receives the same preoperative dose plus three additional intravenous doses of either dicloxacillin (1 g for under 120 kg, 2 g for 120 kg and above) or cefuroxime (750 mg for under 120 kg, 1.5 g for 120 kg and above) within 24 hours after surgery.

The primary outcome is the incidence of PJI within 90 days, defined as a need for revision surgery confirmed by specific microbiological criteria or surgeon report. Data collection for this primary outcome will conclude 90 days after the last patient is enrolled in December 2024. The trial also includes 1-year and 5-year follow-ups to account for PJI that may develop later.

This non-inferiority trial is designed to demonstrate that single-dose antibiotic prophylaxis is non-inferior to multiple doses in preventing PJI following THA.

### ProHipQ-F: single versus multiple dose antibiotic prophylaxis in fracture related THA

This nationwide Danish study aims to determine whether a single preoperative dose of antibiotics is as effective as multiple doses in preventing PJI following primary THA in patients with a fracture or sequelae of proximal femoral or acetabular fractures (22). Similar to the previous study, the trial will include all 36 Danish hospitals performing primary THA, with an expected enrollment of approximately 2,000 patients over a 2-year period.

Patients aged 18 years or older undergoing primary THA due to an acute fracture or sequelae of proximal femoral or acetabular fractures are included. The trial employs a cross-over, cluster randomized design, meaning that each participating center will be randomly assigned to either the single-dose or multiple-dose group for 1 year and then switch to the other group for the following year.

The single-dose group will receive either 3 g of dicloxacillin/cloxacillin or 3 g of cefuroxime intravenously prior to surgical incision. In cases of cephalosporin or general beta-lactam allergy, a single preoperative dose of 900 mg clindamycin will be administered intravenously. The multiple-dose group will receive the same preoperative dose as the single-dose group, followed by three postoperative doses of dicloxacillin/cloxacillin (2 g) or cefuroxime (1.5 g) within 24 hours. After the first postoperative dose, the remaining doses may be administered orally.

The primary outcome of the study is the incidence of PJI, which will be determined based on data from national databases, including the Danish Hip Arthroplasty Register (DHR), the Danish National Patient Registry (DNRP), and the Hospital-Acquired Infections Database (HAIBA). The study will also evaluate secondary outcomes such as serious adverse events, length of hospital stay, and antibiotic use.

The ProHipQ-F trial is designed as a non-inferiority trial, aiming to demonstrate that single-dose antibiotic prophylaxis is not significantly less effective than multiple doses in preventing PJI after THA in patients with a fracture.

### Antibiotic Prophylaxis in Patients Undergoing Elective TKA

This is a multi-center randomized, open-label trial comparing different perioperative antibiotic prophylaxis strategies for preventing PJI and surgical site infections in elective primary total knee arthroplasty (TKA) (NCT03283878). This trial employs a parallel assignment design with two experimental groups (23). Study Group 1 (Single-Dose) patients receive one weight-based dose of prophylactic cefazolin administered intravenously within 60 minutes before skin incision, with no further antibiotic administration. Study Group 2 (Multiple-Dose) patients receive the same preoperative cefazolin dose as Group 1, plus two additional weight-based doses administered within 24 hours postoperatively.

For both groups, dosing is weight-dependent, with patients under 120 kg receiving 2 g of cefazolin and patients 120 kg or above receiving 3 g of cefazolin. Alternative antibiotics such as vancomycin, gentamicin, or clindamycin are permitted for patients with documented penicillin or cephalosporin allergies.

The investigators hypothesize that a single dose of prophylactic antibiotic administered within 60 minutes before incision is not an effective approach to prevent PJI in elective primary TKA. They also hypothesize that prolonged delivery (24 hours) of antibiotic prophylaxis after surgery does not further reduce PJI incidence in elective primary TKA. The primary outcome measured is the rate of periprosthetic infection at 90 days post-operation. Secondary outcomes include PJI rates at 12 months post-operation and costs of antibiotic treatment over a period of up to 1 year.

The study includes patients 18 years and older scheduled for elective TKA for posttraumatic osteoarthritis, avascular necrosis, and/or inflammatory arthritis, without active infections on the operative leg or joint. The trial is led by Duke University and is being conducted across 14 locations in the United States, including medical centers in California, Georgia, Maryland, Mississippi, New Jersey, New York, North Carolina, Ohio, Pennsylvania, South Carolina, Texas, and Virginia.

According to clinicaltrials.gov, the study’s last update was posted in January 2025, suggesting it is still ongoing or has recently completed data collection.

## CONCLUSION

The field of prosthetic joint infection management stands at a pivotal juncture, with an unprecedented wave of clinical research challenging fundamental treatment assumptions and introducing novel therapeutic paradigms. The convergence of large-scale investigator-initiated trials with innovative industry-sponsored studies promises to reshape clinical practice in ways not seen since the establishment of current treatment protocols.

The investigator-initiated trials represent a maturation of PJI research, moving beyond observational studies to address core clinical uncertainties through rigorous randomized controlled designs. The ROADMAP trial’s adaptive platform approach exemplifies this evolution, simultaneously evaluating multiple treatment strategies that have long been debated but never definitively studied. Similarly, the SOLARIO trial’s striking demonstration that prolonged systemic antibiotics may be unnecessary when combined with effective local antibiotic delivery fundamentally challenges current treatment paradigms and offers the potential for significant reductions in antibiotic-related morbidity and healthcare costs.

Novel therapeutic approaches targeting biofilm, including monoclonal antibodies (TRL1068), engineered peptides (PLG0206), and dual-action rifamycin-quinolone hybrid molecules (TNP-2092 and afabicin), show promise in early-phase trials, with staphylococcal species having low MICs with the latter two. However, the translation of these laboratory successes into clinical practice requires demonstration of efficacy in larger populations and cost-effectiveness analyses, particularly in light of the SOLARIO trial results, where systemic antibiotic use for success was not essential beyond the first 7 days. Likewise, even though local antibiotic delivery systems such as VTX are promising, their use in real life will probably be limited due to the complexities around the system and the results of the SOLARIO study.

The prevention studies comparing different doses of prophylactic antibiotics for hip and knee arthroplasties represent one of the largest coordinated investigations of perioperative antibiotic protocols in arthroplasty to date, after the study by Peel et al., where it was demonstrated that the addition of vancomycin to cefazolin prophylaxis was not superior to placebo for the prevention of surgical-site infections in arthroplasty among patients without known MRSA colonization (63). If these current studies can demonstrate that a single dose of antibiotic is sufficient for PJI prevention, the findings will significantly change clinical practice for most centers where antibiotics are routinely continued beyond a single dose.

Several critical considerations emerge from this comprehensive review. The small sample sizes in many novel agent trials limit definitive conclusions about efficacy and safety, while the heterogeneity of PJI as a clinical entity complicates the generalizability of findings across different patient populations and infection scenarios. The financial sustainability of novel therapeutics remains uncertain, as illustrated by the collapse of Phaxiam Therapeutics and its bacteriophage programs, highlighting the significant commercial challenges facing innovative PJI treatments.

Looking forward, the field must prioritize several key research directions. Cost-effectiveness analyses will be essential for novel therapeutics, particularly as healthcare systems face increasing pressure to demonstrate value. Patient stratification strategies are needed to identify which specific populations would benefit most from targeted interventions. The evaluation of combination approaches, integrating both established and novel therapies, may offer synergistic benefits that exceed the efficacy of individual treatments.

The research landscape also reveals important methodological lessons. The success of adaptive platform trials like ROADMAP suggests that this approach may be optimal for addressing multiple clinical questions simultaneously while maintaining statistical rigor. The challenges faced by industry-sponsored trials, including enrollment difficulties and regulatory complexities, underscore the need for collaborative approaches that combine academic expertise with commercial innovation.

Ultimately, the next decade of PJI management will likely be characterized by a more nuanced, evidence-based approach that moves beyond one-size-fits-all protocols toward personalized treatment strategies. The ongoing trials reviewed here represent the foundation for this transformation, offering the potential to improve outcomes while reducing treatment burden for patients facing one of orthopedic surgery’s most challenging complications. The convergence of reduced antibiotic exposure, targeted biofilm disruption, and optimized surgical strategies may finally provide the comprehensive approach needed to tackle the complex pathophysiology of PJI effectively.
